# LncRNA ANRIL is up-regulated in nasopharyngeal carcinoma and promotes the cancer progression via increasing proliferation, reprograming cell glucose metabolism and inducing side-population stem-like cancer cells

**DOI:** 10.18632/oncotarget.11437

**Published:** 2016-08-20

**Authors:** Zhen Wei Zou, Charlie Ma, Lorraine Medoro, Lili Chen, Bin Wang, Roohi Gupta, Ting Liu, Xian Zi Yang, Tian Tian Chen, Ruo Zhen Wang, Wen Jie Zhang, Pin Dong Li

**Affiliations:** ^1^ Cancer Center, Union Hospital, Tongji Medical College, Huazhong University of Science and Technology, Wuhan, 430022, China; ^2^ Department of Radiation Oncology, Fox Chase Cancer Center, American Oncologic Hospital, Pennsylvania, PA 19111, USA; ^3^ Institute of Infection and Immunology, Union Hospital, Tongji Medical College, Huazhong University of Science and Technology, Wuhan, 430022, China; ^4^ Sun Yat-sen University Cancer Center, State Key Laboratory of Oncology in Southern China, Collaborative Innovation Center for Cancer Medicine, Guangzhou, 510060, China; ^5^ Department of Radiation Oncology, Affiliated Tumor Hospital, Xinjiang Medical University, Urumqi, Xinjiang, 830011, China; ^6^ Department of Pathology, Shihezi University School of Medicine, Shihezi, Xinjiang, 832002, China

**Keywords:** nasopharyngeal, carcinoma, LncRNA/ANRIL, mTOR pathway, glucose metabolism

## Abstract

Long noncoding RNAs play a vital role in diverse biological processes such as embryonic development, cell growth, and tumorigenesis. In this study, we report that LncRNA ANRIL, which encodes a 3834-nt RNA that contains 19 exons at the antisense orientation of the INK4B-ARF-INK4A gene cluster, generally up-regulated in nasopharyngeal carcinoma [1]. In a cohort of 88 NPC patients, ANRIL was highly expressed in advanced-stage cancer. Multivariate analyses revealed that ANRIL expression could serve as an independent predictor of overall survival (*P* = 0.027) and disease-free survival (*P* = 0.033). Further investigation showed that knockdown of ANRIL significantly repressed NPC cell proliferation and transformation. We also found that ANRIL could induce the percentage of side population cells (SP cells) in NPC. To meet the urgent needs of energy provision, ANRIL can also reprogram glucose metabolism via increasing glucose uptake for glycolysis, which was regulated by the mTOR signal pathway to affect the expression of essential genes in glycolysis. We concluded that ANRIL could promote NPC progression via increasing cell proliferation, reprograming cell glucose metabolism and inducing side-population stem-like cancer cells. Our results also suggested that ANRIL may serve as a novel diagnostic or prognostic biomarker and a candidate target for new therapies in NPC.

## INTRODUCTION

Nasopharyngeal carcinoma (NPC) is one of the most common head and neck malignancies, presenting regional differences in incidence, such as high occurrences in North Africa, Southeast Asia, and Southern China [2, 3]. Heredity, environmental factors, and the Epstein-Barr virus (EBV) infection play important roles in NPC pathogenesis [4–7]. Although serum anti-EBV antibodies and plasma EBV DNA are very practical for diagnosis and prognosis prediction in NPC, a majority of NPC patients at diagnosis are in advanced stages [8] or present with metastasis [9, 10]. Also, the therapeutic approach for advanced stage individuals remains limited, and the clinical outcome is still unsatisfactory. Furthermore, the precise molecular mechanisms that account for NPC development and progression remain unclear. Therefore, there is still an urgent need to identify novel diagnostic biomarkers for early diagnosis as well as therapeutic targets for NPC patients.

Long noncoding RNAs (lncRNAs) are a major class of transcripts, longer than 200 nt and lack coding-protein potential [11]. Increasing evidence shows that many dysregulated lncRNAs play a vital role in tumor genesis via regulating gene transcription or post- transcriptional regulation [12–14]. Deregulation of LncRNAs was found in multiple tumors where they can act as a tumor suppressor gene or oncogene. LncRNA ANRIL (CDKN2B antisense RNA 1) was initially identified from familial melanoma patients [15]. LncRNA ANRIL encodes a 3834-nt RNA that contains 19 exons at the antisense orientation of the INK4B-ARF-INK4A gene cluster [1]. Since its identification, accumulated research has shown that up-regulation of ANRIL is a risk factor in several human malignancies such as gastric, breast, lung, bladder and ovarian cancer [16–19]. Although ANRIL serves as a fatal oncogene in many cancers, the function of ANRIL in NPC has not been described, and the underlying mechanism remains elusive.

In this study, we are the first to report that ANRIL is up-regulated in NPC. In addition, we found that ANRIL can induce the stem cell-like cells (side population cells, SP cells) in NPC and promote cell proliferation, colony formation, and transformation ability. Further, we found that ANRIL could reprogram the glucose metabolism of NPC cells, which may rapidly provide ATP provision for cell proliferation. All of these factors indicate that ANRIL plays a significant role in the development of NPC.

## RESULTS

### ANRIL is overexpressed in NPC cell lines and NPC biopsy samples

**Figure 1:**
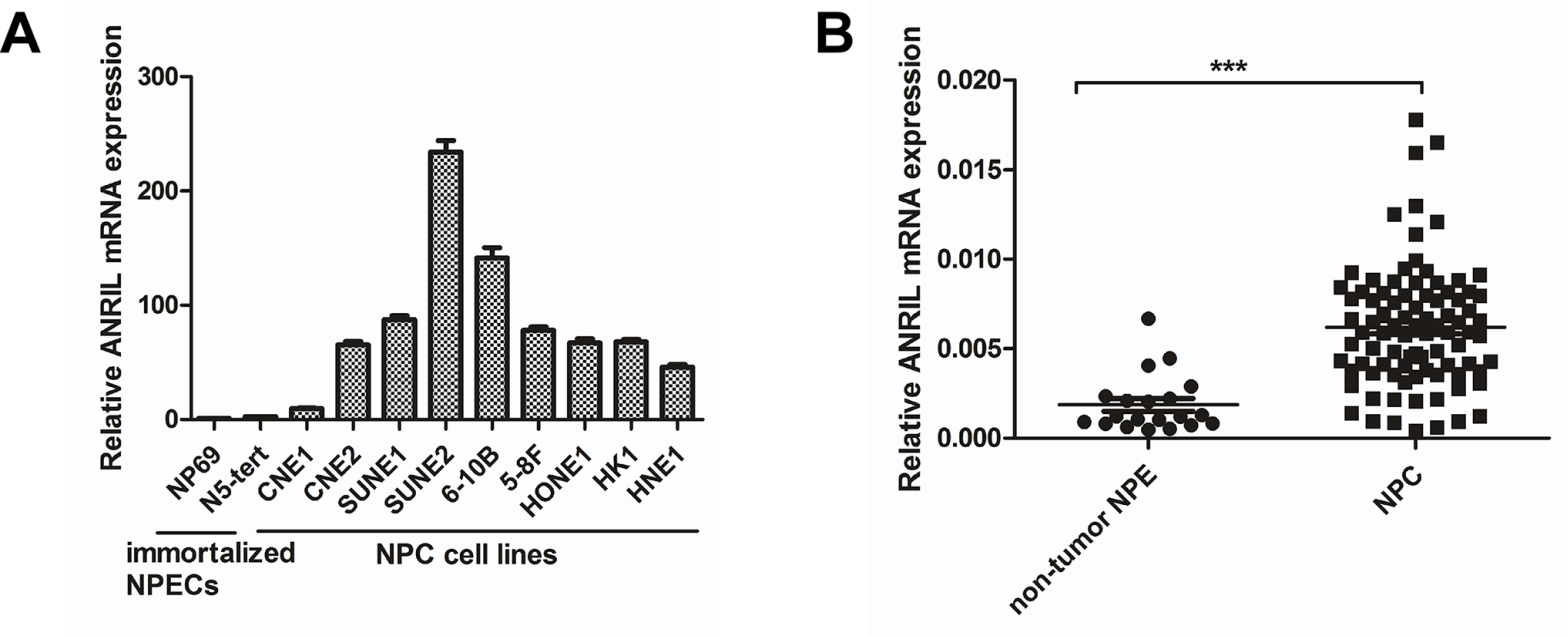
ANRIL expression is frequently upregulated in NPC cell lines and tissues (**A**) Expression of ANRIL was detected by RT-PCR in NPC cell lines and immortalized nasopharyngeal epithelial cells(NPECs). (**B**) Expression of ANRIL was detected by RT-PCR in NPC and non-tumor NPE samples.

To investigate the expression of LncRNA ANRIL in NPC, the qRT-PCR assay was employed in both two immortalized nasopharyngeal epithelial cells (NPECs, N69, and N5-Tert) and a panel of NPC cell lines including CNE1, CNE2, SUNE1, SUNE2, 6-10B, 5-8F, HONE1, HK1 and HNE1. As shown in Figure 1A, the expression LncRNA ANRIL was barely detectable in NP69 and N5-Tert, whereas a much higher expression in NPC cells was noted. Furthermore, we performed qRT-PCR in eighty-eight NPC biopsy samples and twenty non-tumor NPE biopsies to identify whether ANRIL was also overexpressed in NPC patients. Consistently, the expression of ANRIL in NPC tissues was significantly higher than the level in non-tumor NPE biopsies (Figure 1B). In conclusion, these data demonstrated that LncRNA ANRIL was frequently upregulated in both NPC cell lines and tissues.

### Relationship between ANRIL expression and progression of NPC or patient survival

**Figure 2:**
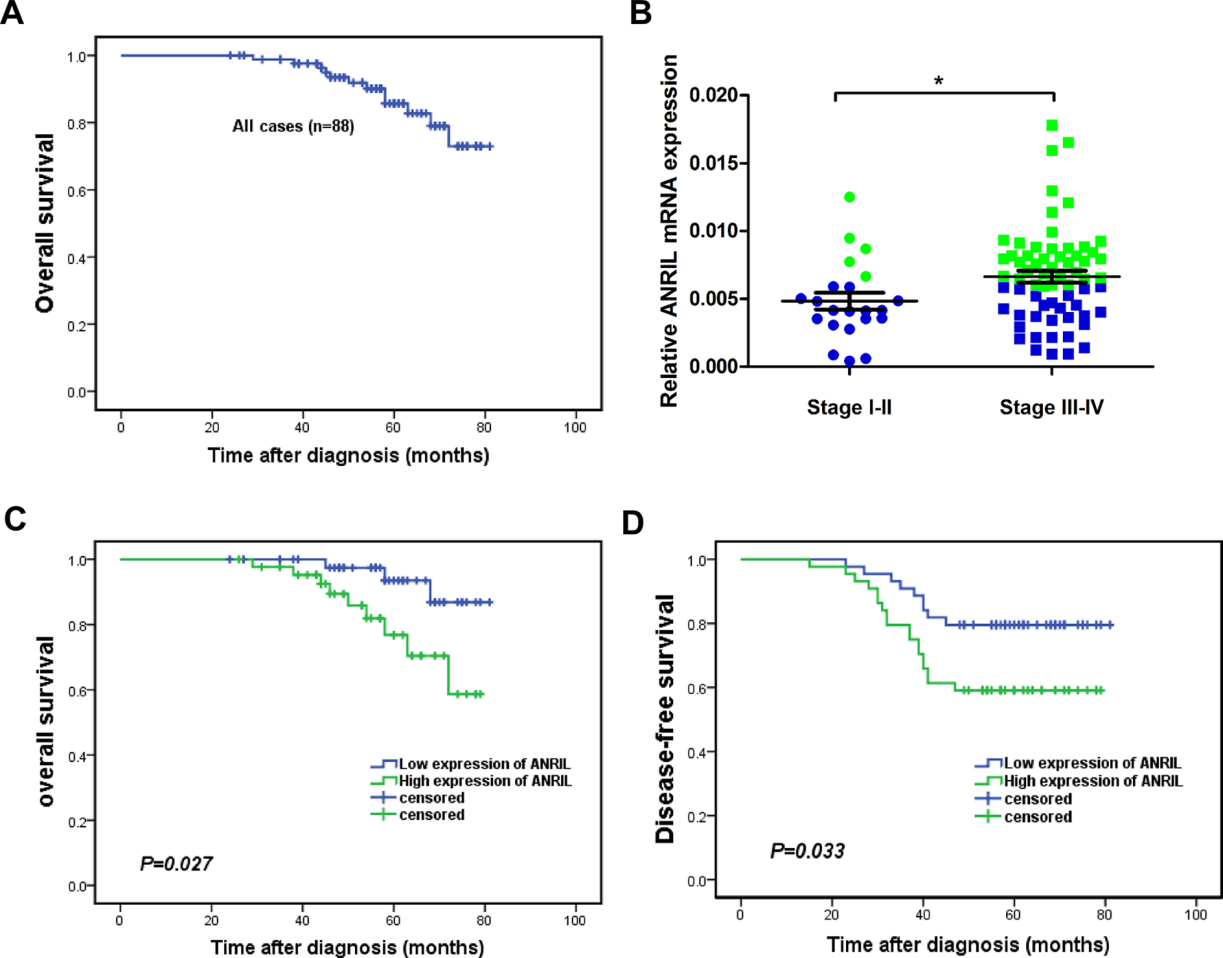
High expression of ANRIL indicated a short overall survival and disease-free survival in NPC patients (**A**) The 5-year overall survival (OS) rate was 85.7% for the total study population. (**B**) The expression of ANRIL increased in advanced stages of NPC (**C**) Kaplan-Meier survival analysis of ANRIL expression for overall survival (**D**) Disease-free survival curves of patients with low and high ANRIL expression.

To reveal the potential role of ANRIL in NPC, we further analyzed the relationship between ANRIL expression and clinicopathological characteristics of the 88 NPC patients in this study. The 5-year overall survival rate was 85.7% for the total study population (Figure 2A). We defined the median expression of ANRIL from 88 NPC tissue samples as the cut-off value. The expression of ANRIL was high when the value more than cut-off value, otherwise low expression. As presented in Table 1, ANRIL expression correlated with the clinical stage (*p* = 0.003) and was higher in stage III–IV patients compared with stage I–II patients and high expression samples were presented as green dots, and low expression samples, as blue dots (Figure 2B). In addition, ANRIL was also associated with locoregional recurrence (*p* = 0.037). However, there was no significant relationship between patient's age, sex, T classification, N classification or distant metastasis. We then used the Kaplan-Meier test to analyze the correlation between the expression of ANRIL and patient survival. From this analysis, we found that patients with low ANRIL expression had longer overall survival (*p* = 0.027, Figure 2C). Consistently, disease-free survival (PFS) was shorter for patients with high ANRIL expression than those with low ANRIL expression (*p* = 0.033, Figure 2D).

**Table 1:** Correlation between the expression of ANRIL and clinicopathologic features in NPC

Characteristics	No. of patients	Expression of ANRIL		*P* value^a^
		Low	High	
Patients	88	44	44	
Age at diagnosis				
Median	48			
Range	21~82			
≤ 48	45	21	24	0.522
> 48	43	23	20	
Sex				
Female	18	12	6	0.113
Male	70	32	38	
T classification				
T1–T2	50	28	22	0.197
T3–T4	38	16	22	
N classification				
N0–N1	37	21	16	0.280
N2–N3	51	23	28	
Clinical stage				
I–II	22	17	5	0.003
III–IV	66	27	39	
Distant metastasis				
Yes	31	16	15	0.823
No	57	28	29	
Locoregional recurrence				
Yes	27	9	18	0.037
No	61	35	26	

a Chi-square test.

Next, we performed univariate and multivariate analyses using the COX proportional hazards model to determine whether ANRIL expression could serve as an independent prognostic predictor. A series of factors, including patient's age, gender, T classification, N classification, clinical stage, distant metastasis, and ANRIL expression level, were performed in the univariate Cox regression analysis to test their association with the OS and PFS of NPC patients. As shown in Table 2, ANRIL was indeed a prognostic factor for OS and PFS. Additionally, the T classification, N classification and clinical stage were prognostic factors for OS, while N classification was also a prognostic factor for PFS. The variables most significantly associated with OS or PFS in the univariate analysis were further analyzed by multivariate analysis. The multivariate analysis model revealed predominantly independent predictors of OS and PFS were ANRIL expression and the N classification as shown in Table 3.

**Table 2:** Univariate analysis with the COX proportional hazards for predictor of OS and PFS of NPC patients

Prognostic factors	*p*	HR	95% CI for HR	
			Lower	Upper
OS				
Age (years) (> 48 vs. ≤ 48)	0.414	0.618	0.195	1.961
Sex (female vs. male)	0.359	0.384	0.049	2.976
T classification (T3–4 vs. T1–2)	0.041	4.900	1.070	22.433
N classification (N2–3 vs. N0–1)	0.035	5.158	1.124	23.675
Clinical stage (III–IV vs. I–II)	0.017	2.930	1.338	6.117
ANRIL(High vs. low)	0.041	3.919	1.057	14.526
DFS				
Age (years) (> 48 vs. ≤ 48)	0.839	0.925	0.434	1.967
Sex (female vs. male)	0.737	1.168	0.471	2.894
T classification (T3–4 vs. T1–2)	0.098	0.498	0.218	1.137
N classification (N2–3 vs. N0–1)	0.001	7.622	2.292	25.350
Clinical stage (III–IV vs. I–II)	0.198	0.591	0.266	1.316
ANRIL (High vs. low)	0.040	2.316	1.040	5.158

**Table 3:** Multivariate analysis with the COX proportional hazards for the predictor of OS and DFS of the NPC patients

Prognostic factors	*p*	HR	95% CI for HR	
			Lower	Upper
OS				
Age (years) (> 48 vs. ≤ 48)				
Sex (female vs. male)				
T classification (T3–4 vs. T1–2)	0.032	5.906	1.170	29.818
N classification (N2–3 vs. N0–1)	0.074	4.169	0.870	19.981
Clinical stage (III–IV vs. I–II)	0.206	0.242	0.027	2.184
ANRIL(High vs. low)	0.018	4.340	2.691	27.268
DFS				
Age (years) (> 48 vs. ≤ 48)				
Sex (female vs. male)				
T classification (T3–4 vs. T1–2)				
N classification (N2–3 vs. N0–1)	0.001	7.210	2.165	24.018
Clinical stage (III–IV vs. I–II)				
ANRIL (High vs. low)	0.012	2.062	1.625	4.598

### Knockdown of ANRIL expression suppresses the proliferation and clonogenicity of NPC cells

**Figure 3:**
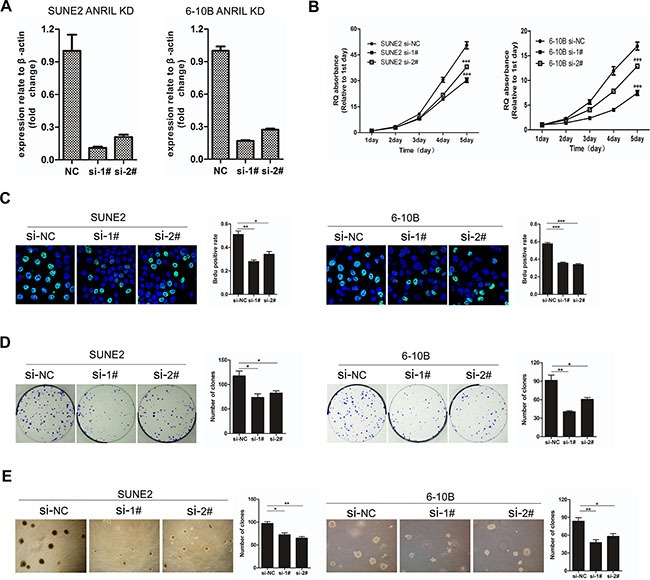
Knockdown of ANRIL suppressed the proliferation and transforming ability of NPC cells (**A**) Knock-down of ANRIL by different siRNAs resulted in reduced ANRIL mRNA expression in SUNE2 and 6-10B cell lines. (**B**) The MTT assay measured the viability of SUNE2 and 6-10B cells infected with NC or ANRIL-targeting siRNAs. (**C**) Brud assay of SUNE2 and 6-10B cells infected with NC or ANRIL-targeting siRNAs. This assay was performed in triplicate (left panel). The percentage of Brud positive cells was shown in right panel. (*P < 0.05, **P < 0.01, ***P < 0.001 by the paired *t*-test) (**D**) Colony-formation assay of SUNE2 and 6-10B cells infected with NC or ANRIL-targeting siRNAs. This assay was performed in triplicate (left panel); the quantification of colony formation shown in right panel. (*P < 0.05, **P < 0.01, ***P < 0.001 by the paired *t*-test) (**E**) The anchorage-independent growth in soft agar of SUNE2 and 6-10B cells infected with NC or ANRIL-targeting siRNAs. This assay was performed in triplicate (left panel); the quantification shown in right panel. (*P < 0.05, **P < 0.01, ***P < 0.001 by the paired *t*-test).

To explore the potential role of ANRIL in NPC tumorigenesis, we used two independent siRNAs to knock down ANRIL expression in 6-10B and SUNE2 cells. The different transcripts of ANRIL and the siRNA targeted site were shown in Supplementary Figure S1A. A significant reduction of ANRIL was identified by qRT-PCR (Figure 3A). It is reported that expression of ANRIL has been correlated with expression of other genes/transcripts in the ARF cluster (p14/ARF, p15 and p16) [20]. In our work, we also detected the expression of p14, p15 and p16 in NPC cells. Interestingly, we found that the expression of p14 and p15 were all up-regulated when knock-down of ANRIL in 6-10B and SUNE2 cells, while the expression of p16 had no significant difference when knock-down of ANRIL those tow cells (Supplementary Figure S1B). We next performed the MTT assay to test the effect of ANRIL knocking down cell proliferation. The results showed that siANRIL treatments caused markedly lower tumor cell viability, compared with the negative control (NC) treatment (Figure 3B). In addition, as shown in BrdU assay (Figure 3C), the percentage of BrdU-positive cells decreased after siANRIL treatments. We also found that knockdown of ANRIL in 6-10B or SUNE1 cells resulted in dramatically fewer and smaller colonies compared to the NC group (Figure 3D). Anchorage-independent growth assay (Soft-agar assay) showed that ANRIL could enhance the transformation ability of NPC cells (Figure 3E). All of these results demonstrated that LncRNA ANRIL could function as an oncogene in NPC, which can promote cell proliferation and enhance the transforming ability of NPC cells.

### Knockdown of ANRIL attenuated cancer stem cell-like properties in NPC cells

**Figure 4:**
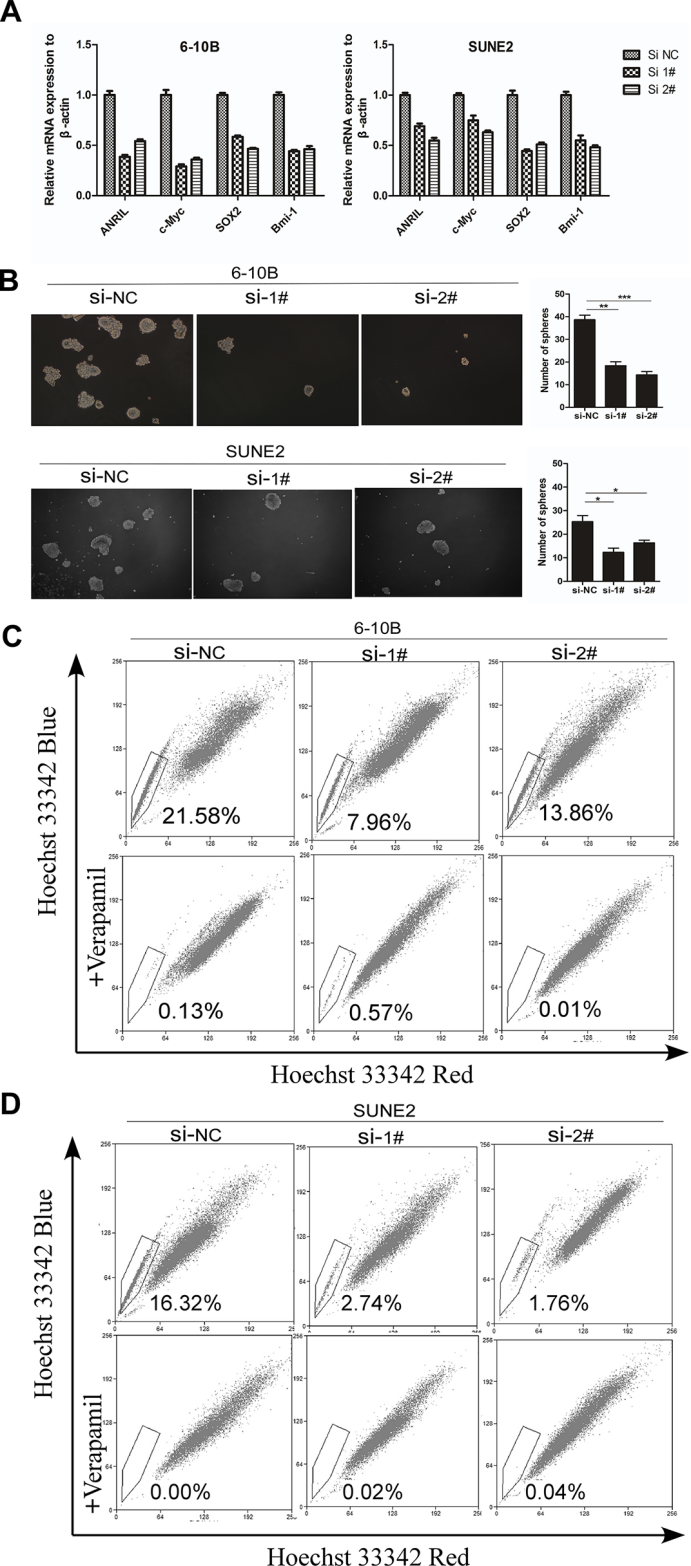
ANRIL silencing suppressed the percentage of side-population cells in NPC cells (**A**) Real-time PCR analysis of mRNA expression of pluripotency-associated markers in SUNE2 and 6-10B cells after ANRIL knock-down. (**B**) Representative micrographs (left panel) and quantification (right panel) of tumor sphere formation in ANRIL knock-down cells (**C**) Knock-down of ANRIL decreased the percentage of the side-population (SP) cells. Flow cytometric patterns of SP cells in 6-10B cell after ANRIL silence. SP cells profiles in the presence of verapamil are shown in the bottom panels. The percentages of SP cells are indicated. (**D**) Knock-down of ANRIL decreased the proportion of the side-population (SP) cells. Flow cytometric profile of SP cells in SUNE2 cell after ANRIL silence. SP cells profiles in the presence of verapamil are shown in the bottom panels. The percentages of SP cells are indicated.

Recently, it has been reported that depletion of ANRIL markedly reduced the proliferation of human colorectal cancer cells in three-dimensional cultures [21]. We postulate that ANRIL expression may reduce cancer stem cells in NPC, which could contribute to the oncogenic function. As reported, c-Myc, SOX2, and Bmi-1 could well represent the stem cell markers in NPC cells [22, 23]. Then to determine whether knockdown of ANRIL could decrease such stem cell-like phenotypes in NPC, representative stem cell markers were analyzed by Q-PCR. As expected, we observed that siANRIL treatments could naturally reduce the stem cell markers c-Myc, SOX2, and Bmi-1 at the RNA level (Figure 4A). Cancer stem cells have the ability to grow forming suspended spherical, clonal colonies, and the tumor sphere-forming assay has been widely used in many stem cell types of research [24]. The additional findings from tumor-sphere formation assay showed that knockdown of ANRIL formed less and smaller spheres than negative control cells (Figure 4B). As we all know, side populations (SPs) in NPC cells have been reported to exhibit cancer stem cell characteristics [23]. The side-population (SP) cells defined by the higher efflux of DNA-binding dye Hoechst 33342 [25, 26]. To identify whether ANRIL expression may contribute to the induction of SP cells in NPC, we performed Hoechst 33342 flow cytometry to detect the number of SPs in NPC cells after ANRIL silence. A decreased percentage of SPs was observed in the siANRIL treatment groups (Figure 4C and 4D), in the meantime, verapamil (a specific inhibitor of ABCG2) apparently inhibited the efflux of Hoechst 33342. All of these results suggested that ANRIL may stimulate the stem cell properties of NPC which may contribute to cell proliferation.

### Knockdown of ANRIL reprogramed the glucose metabolism of NPC cells via mTOR signal

**Figure 5:**
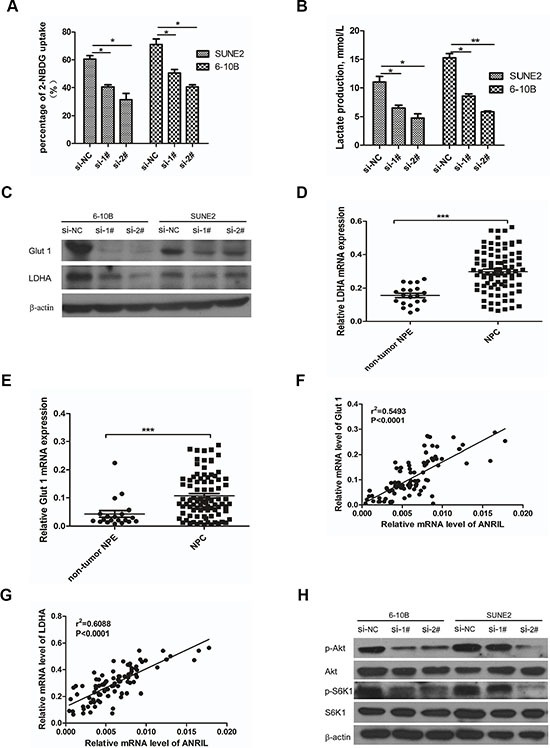
Knockdown of ANRIL reprogramed the glucose metabolism of NPC cells via mTOR signal (**A**) SUNE2 and 6-10B were grown in the presence of the fluorescent glucose analog (2-NBDG) for 1hr after ANRIL knock-down, and the glucose uptake was then quantified using flow cytometry. This assay was performed in triplicate (*P < 0.05, **P < 0.01, ***P < 0.001 by the paired *t*-test). (**B**) The production lactate of SUNE2 and 6-10B after ANRIL knock-down detected by Lactate Assay Kit (BioVision). This assay was performed in triplicate (*P < 0.05, **P < 0.01, ***P < 0.001 by the paired *t*-test) (**C**) Knock-down of ANRIL in SUNE2 and 6-10B cells decreased the expression of Glut 1 and LDHA in protein level (**D**) The expression of Glut 1 in NPC tissues was up-regulated. (**E**) The expression of LDHA in NPC tissues was up-regulated. (**F**) The association between ANRIL and Glut 1 in 88 NPC tissues was performed. (**G**) The association between ANRIL and LDHA in 88 NPC tissues was performed. (**H**) mTOR signal pathway in SUNE2 and 6-10B cell lines was inactivated after ANRIL silence.

As demonstrated above, ANRIL could induce stem cell-like phenotypes (side-populations) in NPC, and further, contribute to uncontrolled cell proliferation that may represent the essence of NPC. So, corresponding adjustments of energy metabolism must be gained to fuel cell growth and division [27]. Given that cancer cells often reprogramed metabolism to cope with the demand of cell mass increase during growth, we next examined whether the ANRIL involved the metabolic reprogramming of NPC cells. First, it was necessary to review whether ANRIL could influence glucose uptake, the primary source of fuel for cancer cells. Flow cytometry was employed to detect the glucose uptake in the siANRIL treatment groups and NC treatment groups using a fluorescent glucose analog (2-NBDG) which was incubated with cells and allowed quantification of glucose uptake [28]. Notably, siANRIL treatments displayed a striking decrease in glucose uptake after 1 hr following the addition of the glucose analog (Figure 5A). It is reported that cancer cells can reprogram their glucose metabolism by limiting their energy metabolism largely to glycolysis, even in the presence of oxygen. This aerobic glycolysis will result in increased lactate production [29]. Next, we measured the lactate production, to determine whether aerobic glycolysis was impaired after ANRIL knockdown. Indeed, ANRIL silence lead to significantly lower levels of lactate production when compared to NC treatment cells (Figure 5B). Next, we sought to assess how ANRIL affects glucose utilization. Then, we attempted to determine whether the decreased glucose uptake and lactate production were associated with the expression of glucose transporters and enzymes that convert pyruvate to lactate. Glut 1 is the primary glucose transporter that modulates the basal uptake of glucose, while LDHA is the key enzyme that catalyzes the conversion pyruvate to lactate in the final step of aerobic glycolysis [30, 31]. Therefore, we employed the western blot to detect the expression of Glut 1 and LDHA in ANRIL knockdown cells. We found that ANRIL knockdown cells expressed substantially lower levels of Glut 1 and LDHA (Figure 5C), consistent with the decreased glucose uptake and lactate production in these cells. For further analysis of the relationship between ANRIL and Glut 1 or LDHA, we also evaluated the expression of Glut 1 and LDHA in the eighty-eight NPC biopsy samples and twenty non-tumor NPE biopsies. We observed that both Glut 1 and LDHA exhibited significantly higher expression in NPC tissues than that in non-cancerous tissues (Figure 5D and 5E). And we also found that the expression of Glut 1 and LDHA correlated with ANRIL (Figure 5F and 5G). As previously shown, ANRIL could control gastric cancer cell proliferation by regulating the mTOR pathway [16]. The mTOR signal pathway plays an important role in glucose metabolism [32]. To investigate the mTOR signal activation in NPC cells in our current study, western blot analysis using antibodies that detect S473 phosphorylation of Akt and T398 phosphorylation of S6K1 was performed to detect the activation of mTOR. As shown in Figure 5H, phosphor-Akt (S473) and phosphor-S6K1 (T398) were found to be down-regulated after ANRIL knockdown. Taken together, these data demonstrated that ANRIL could increase the expression of Glut1 and LDHA in NPC cells. Both ANRIL and Glut1 or LDHA can contribute to NPC progression. The possible mechanism of how ANRIL regulating the expression of Glut1 and LDHA may be that ANRIL promotes the phosphorylation of Akt to activate the mTOR signal pathway, which can up-regulate the expression of Glut1 and LDHA. Subsequently, the glucose uptake would be enhanced, and the increased glucose could be reprogrammed to aerobic glycolysis for rapid ATP production for proliferation.

## DISCUSSION

Nasopharyngeal carcinoma (NPC) is one of the most common head and neck malignancies in Southern China and Southeast Asia [2, 3]. Its development was associated with genetic factors, environmental factors, and the Epstein-Barr virus (EBV) infection [4–7] [17]. More than 60% of NPC patients are in advanced stages at diagnosis or even with metastasis. Therefore, there is still an urgent need to identify novel diagnostic biomarkers for early diagnosis as well as therapeutic targets for NPC patients. The targeted therapy will provide new insight into its pathogenesis.

To date, increasing evidence shows that many dysregulated lncRNAs play a vital role in tumor genesis by regulating gene expression at transcriptional or post- transcriptional levels. Many studies have recently focused on the function of ANRIL in different cancers. As reported, ANRIL plays a a critical role in tumorigenesis in gastric, liver, lung, bladder, ovarian and other cancers [21, 33–38]. Previous studies have demonstrated that the mechanism for ANRIL's function was epigenetic regulation of p15^INK4B^ and p16^INK4A^ in Cis by binding to PRC2 [39, 40]. However, to the best of our knowledge, the function of LncRNA ANRIL in NPC is still limited. The results of our current study identified that LncRNA ANRIL plays a pivotal role in the progression of NPC. We first demonstrated the novel functions of ANRIL, which can promote cell proliferation and transformation via stem-like cancer cell induction. To meet the urgent needs of energy provision, ANRIL can also reprogram glucose metabolism via increasing glucose uptake for glycolysis to produce more ATP, which was regulated by the mTOR signal pathway.

Cancer stem cells (CSCs), a population of cancer cells that possess the ability of proliferation and transformation, are thought to be responsible for tumor initiation and progression [41, 42]. Recently, collective studies have demonstrated that LncRNA played a significant role in the production of CSCs, which contribute to tumor genesis and progression. For example, Lu et al. showed that LncRNA CUDR promoted liver cancer stem cell growth through up-regulating Tert and C-myc [43]. As reported that stem cell-like side population cells in NPC indicated a poor prognosis [44–46]. In our study, we found that knockdown of ANRIL in NPC cell lines could decrease the percentage of side population cells. Consistently, the clinically significant finding suggested that high ANRIL expression in NPC was associated with shorter survival time and more frequent loco-regional recurrence.

Distinct from normal cells, cancer cells acquire alterations in central metabolic pathways to fulfill their high demands on biomass and energy production, while maintaining appropriate redox [47]. These metabolic changes are critical for cancer cells to sustain rapid proliferation and adapt to a dynamic tumor microenvironment [48, 49]. Increasing LncRNAs were found to be involved in metabolic regulation. For an example, Hung et al. demonstrated that LncRNA PFGEM1 could serve as a key transcriptional regulator of central metabolic pathways in prostate cancer cells and connect c-Myc to tumor metabolism [50]. It is reported that ANRIL could regulate key genes of glucose and fatty acid metabolism in several diseases [51, 52]. Consistently, in the present study, we identified that knockdown of ANRIL could decrease the expression of Glut 1 and LDHA, which were essential genes in glycolysis metabolism. Besides, in clinical data, we found a strong positive relationship between ANRIL and Glut 1 or LDHA. The mechanism of ANRIL regulating Glut 1 and LDHA expression may be at both transcriptional and post-transcriptional levels. However, we demonstrated that the mTOR signal pathway, which is important in glucose metabolism, could be affected by the expression of ANRIL. We postulate that ANRIL contributes to the increased expression of essential genes in glucose metabolism, which would account for the reprogrammed glucose metabolism of cancer cells.

In summary, our clinical data indicated that ANRIL was highly expressed in NPC, which was correlated with clinical stage, and could serve as an independent predictor for OS and DFS. Moreover, lossoffunction studies demonstrated that ANRIL promoted NPC cell proliferation and transformation, and this oncogenic function may be attributed to the induction of NPC stem cells (side population cells, SP cells). Finally, we demonstrated that ANRIL increased Glut 1 and LDHA expression to reprogram the glucose metabolism of NPC cells, which may partially account for ANRILinduced NPC SP cells and tumorigenesis. We also identified that these biological processes may be regulated by the mTOR signal pathway. Our study is the first to clarify that ANRIL promotes NPC progression via increasing cell proliferation, reprograming cell glucose metabolism and inducing side-population stem-like cancer cells. Importantly, ANRIL may be a novel diagnostic or prognostic biomarker and a potential therapeutic target for NPC treatment.

## MATERIALS AND METHODS

### Patients and tissue specimens

Eighty-eight NPC biopsy samples and twenty non-tumor NPE biopsies were acquired from Cancer Center of Union Hospital, Tongji Medical College, Huazhong University of Science and Technology; Wuhan China was used to verify ANRIL expression with qRT-PCR. All tissue samples were collected from newly diagnosed NPC patients from 2009 to 2012 and confirmed by histopathological examination. Tissues were obtained from biopsy, immediately immersed in the RNA-Later reagent overnight at 4°C, and stored at −80°C before RNA extraction. This study was approved by the Institutional Research Ethics Committee at the Cancer Center. The clinical characteristics were collected from patient medical records and were described in Table 1.

### Cell culture

Normal immortalized human nasopharyngeal epithelial cells (NP69 and N5-Tert) were cultured in Keratinocyte serum-free medium (Invitrogen, Carlsbad, CA, USA). The NPC cell lines (CNE2, CNE1, SUNE1, HONE1, HK1, S26, S18, 5-8F, 6-10B, and HNE1) were maintained in Roswell Park Memorial Institute (RPMI)1640 medium (Invitrogen) supplemented with 5% fetal bovine serum (FBS; Hyclone, Logan, UT) in a humidified 5% CO2 incubator at 37°C.

### RNA extraction and reverse transcription

Each tissue was transferred to a liquid nitrogen-precooled mortar and ground into a powder using a pestle. Then total RNA was extracted from the tissue specimens and NPC cell lines using the TRIzol reagent (Invitrogen), according to the manufacturer's instructions. Approximately 1 μg total RNA was reverse-transcribed using the ThermoScript reverse transcription-Polymerase Chain Reaction (RT-PCR) system (Invitrogen,), based on the manufacturer's instructions.

### Reverse transcription-PCR (RT-PCR)

qRT-PCR was performed using the LightCycler 480 SYRB Green I MasterMix (Roche, Indianapolis, USA) to detect the level of ANRIL using a LightCycler 480 II (Roche, Basel, Swiss). β-actin was used as an internal control. The relative expression of ANRIL was normalized to the expression of β-actin, which yielded a 2-Δct value. Quantitative determination of the RNA levels was performed in triplicate in three independent experiments. The sequences of the real-time PCR primers were as follows:

ANRIL -sense: 5′- TGCTCTATCCGCCAATCAGG -3′,

ANRIL - antisense: 5′- GGGCCTCAGTGGCACATACC -3′

c-Myc -sense: 5′- GCGTCCTGGGAAGGGAGATC-3′,

c-Myc - antisense: 5′- GGGCATCGTCGCGGGAGGCTG-3′

SOX2 -sense: 5′- CGAGTGGAAACTTTTGTCGGA -3′,

SOX2 - antisense: 5′- TGTGCAGCGCTCGCAG-3′

Bmi-1 -sense: 5′- CTGGTTGCCCATTGACAGC-3′,

Bmi-1 - antisense: 5′- CAGAAAATGAATGCGAGCCA-3′

Glut 1 -sense: 5′- TTGGCTCCGGTATCGTCAAC-3′,

Glut 1 - antisense: 5′- GGCCACGATGCTCAGATAGG-3′

LDHA -sense: 5′- AGCCCGATTCCGTTACCT-3′,

LDHA - antisense: 5′- CACCAGCAACATTCATTCCA-3′

β-actin sense: 5′-CGCGAGAAGATGACCCAGAT-3′

β-actin antisense: 5′-GGGCATACCCCTCGTAGATG-3′

### siRNA transfection

The siRNAs targeting the LncRNA of human ANRIL [Gene ID: 100048912, NR_003529.3] were denoted as siANRIL-#1 (GGUCAUCUCAUUGCUCUAU) and siANRIL-#2 (GCCCAAUUAUGCUGUGGUA). The negative control (NC) was indicated as siNC which was nonhomologous to any human genome sequence. The 6-10B and SUNE1 cells were plated in 6-well plates for 18 h and then transfected with 20 nM of the RNA duplex and 5 μL of Lipofectamine RNAiMAX (Invitrogen), according to the manufacturer's instructions. Cells were cultured for 48 h and harvested for further experimentation.

### Western immunoblotting

Cells were harvested and lysed in SDS sample buffer (62.5 mM Tris-HCl (pH 6.8), 3% sodium dodecyl sulfate (SDS), 10% glycerol, 50 mM DL-dithiothreitol (DTT), and 0.1% bromophenol blue) with protease inhibitors (Roche, Indianapolis, IN, USA). The protein concentrations were determined by the BCA method (Pierce, Thermo Fisher Scientific Inc., Rockford, IL, USA). The proteins (30 μg) were separated by SDS-PAGE and transferred to a polyvinylidene difluoride membrane. Bovine serum albumin (5%) in TBS-T (1 mol/L Tris-HCl (pH 7.5), 0.8% NaCl and 0.1%Tween 20) was used to block the membrane. Then, the membrane was incubated with anti-Glut1 (Santa-Cruz, sc-7903, CA, USA), anti-LDHA (Santa-Cruz, sc-137243, CA, USA), anti-phosphor-S6K1 (Cell Signaling, #9209) and total S6K1 (Cell Signaling, #9202), anti-phosphor-AKT (Cell Signaling, #13038) and total Akt (Cell Signaling, #4691) and anti-β-actin (Sigma-Aldrich, A5441, St.Louis.USA) antibodies at 4°C overnight. The blots were then treated with an HRP-conjugated secondary antibody (Pierce)

### 3-(4,5-Dimethylthiazol-2-yl)-2,5-diphenyltetrazoliumbromide (MTT) assay

The MTT assay was used to measure the viability of the NPC cells. Cells transfected with specific siRNA were seeded onto a 96-well plate at a density of 1500 cells/well. Each sample had six replicates. At the indicated time points, cells were added to 20 μL of 5 mg/mL MTT and incubated for four h at 37°C. Then 200 μl DMSO/well was added to the culture cells to dissolve the crystals, and the cells were counted daily by reading the absorbance at 490 nm using the Spectramax M5 (Molecular Devices, Sunnyvale, USA).

### Colony-formation assay

After transfection with the specific siRNAs, Cells (400 cells per well) were plated evenly in 6-well plates and cultured for 14 days. The clones were washed twice with PBS, fixed in methanol for 15 min, and stained with 0.5% crystal violet in 20% methanol for 15 min at room temperature. After removing the dye by washing, the plates were photographed and quantified. At least three independent experiments were carried out for each assay.

### Soft agar assays

Soft agar was prepared by mixing equivalent volumes of 1% agarose in PBS with the 2×fresh medium; the mixture was added to each well of a 6-well plate and kept at 4°C for 15 minutes. Cells were prepared in 0.3% agar and seeded in triplicate 6000 cells/well. The plates were then incubated at 37°C in a humid atmosphere of 5% CO2 for two weeks until colonies had formed. Each experiment was repeated at least three times. Colonies were photographed between 18–24 days (final magnification 40 ×) under a phase contrast microscope and colonies were counted under the microscope.

### Tumor sphere formation assays

48 hours after transfection with specific siRNAs, cells (100 cells per well) were seeded in 6-well ultra-low cluster plates for ten days. Spheres were cultured in DMEM/F12 serum-free medium (Invitrogen, San Diego, CA, USA) supplemented with 2% B27 (Invitrogen, San Diego, CA, USA), 20 ng/ml EGF, 20 ng/ml bFGF, and 5 μg/ml insulin (Invitrogen, San Diego, CA, USA).

### Detection of the side population of NPC cell lines

48 hours after transfection with specific siRNAs, 6-10B, and SUNE1 cells were trypsinized and resuspended at a density of 1 × 10^6^ cells/ml. The DNA binding dye, Hoechst 33342 (Sigma-Aldrich), was then added at a final concentration of 5 μg/ml and incubated for 90 min in the dark with every half an hour mixing. Then the cells were washed with PBS for twice. The cells were placed at 4°C in the dark before flow cytometry (EPICS ALTRA Flow Cytosorter, Beckman Coulter) using dual wavelength analysis. While, a portion of the cells was incubated with100ug/ml verapamil (a calcium ion tunnel antagonist, Sigma-Aldrich) for 30 min at 37°C prior to adding Hoechst 33342 to determine whether this would block the fluorescent efflux of SP cells in the 6-10B and SUNE1 populations.

### Glucose uptake assay and lactate production assay

For glucose uptake assays in cells, cells were transfected with specific siRNA for about 36 hours, then 10 uM 2-NBDG (Invitrogen) was added to the media for two hr. After incubation, flow cytometry was performed to detect the percentage of glucose uptake. To measure lactate production, specific siRNA transfected cells in 80% confluent were replenished with fresh medium. After 24 hours, lactate concentration was determined with the Lactate Assay Kit (BioVision). Optical density (OD) was measured at 570 nm, 30 min after the addition of substrate.

### Statistical analysis

Data were analyzed by the SPSS standard version 17.0 (SPSS, Chicago, USA) and GraphPad Prism version 5.0 (GraphPad Software, San Diego, CA, USA). The Kaplan–Meier method was used to estimate overall survival (OS) and multivariate analysis was performed by the Cox proportional hazards model. The chi-square test was used to analyze the correlation between the clinical features and ANRIL expression. Analysis of the differences between groups was determined with the two-tailed Mann–Whitney test. Data were presented as the mean ± standard error of the mean (SEM) obtained from three independent experiments. *P* values less than 0.05 were considered statistically significant.

## SUPPLEMENTARY MATERIAL FIGURE


