# Supplementary material for: LncRNA ANRIL is up-regulated in nasopharyngeal carcinoma and promotes the cancer progression via increasing proliferation, reprograming cell glucose metabolism and inducing side-population stem-like cancer cells
Source: Oncotarget. 2016 Aug 20;7(38):61741–54. doi: 10.18632/oncotarget.11437 (PMC5308687; doi:10.18632/oncotarget.11437)
Supplement: Supplementary file 1 [file oncotarget-07-61741-s001.pdf]

LncRNA ANRIL is up-regulated in nasopharyngeal carcinoma and promotes the cancer progression via increasing proliferation, reprograming cell glucose metabolism and inducing side-population stem-like cancer cells

Supplementary Materials

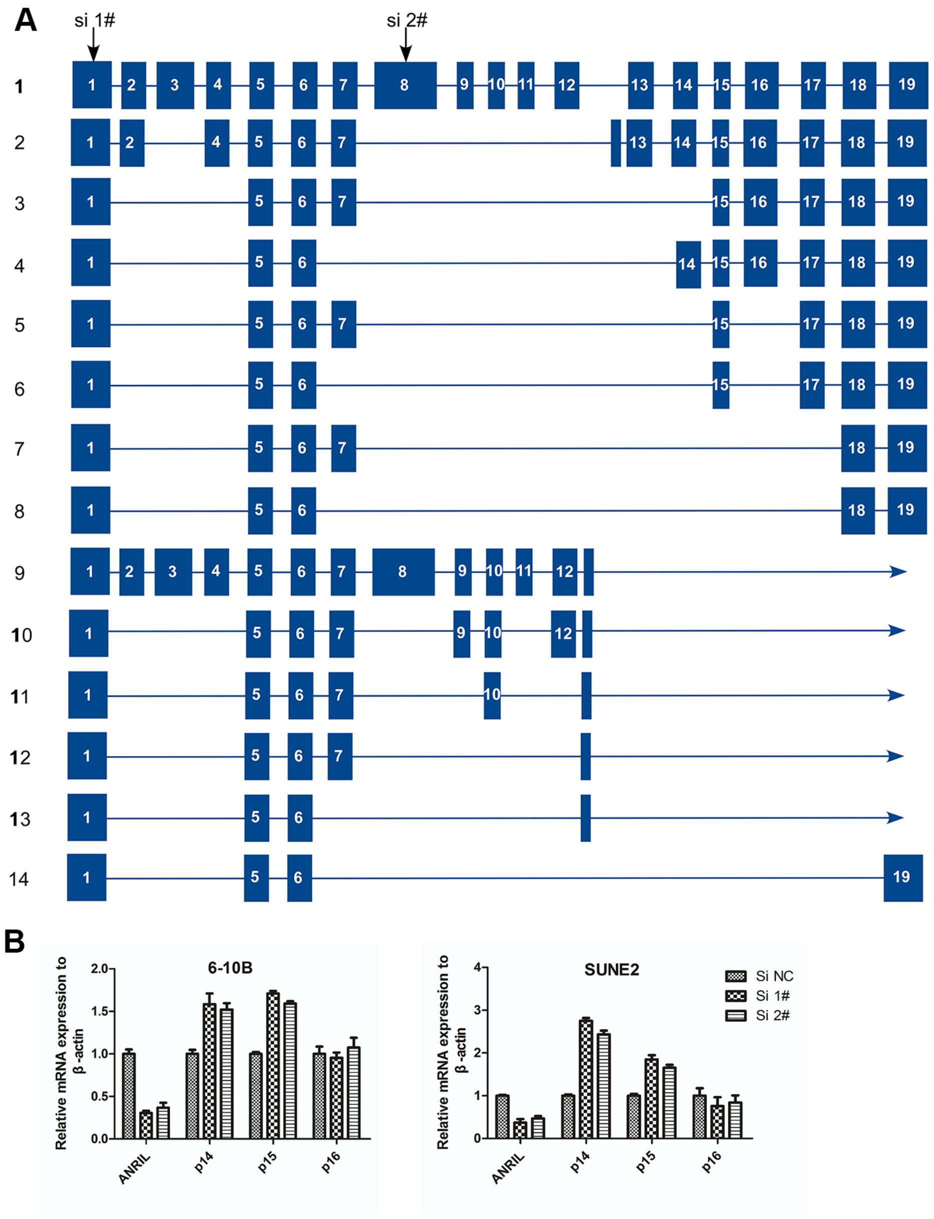

Supplementary Figure S1: ANRIL enjoyed multiple transcript isoforms and affected the INK4B-ARF-INK4A gene cluster expression. (**A**) Diagrammatic representation of the ANRIL gene and the 14 transcripts. The tow arrows showed the targeted site of siRNAs for ANRIL. (**B**) Knock-down of ANRIL in SUNE2 and 6-10B cells increased the expression of p14 and p15 in RNA level, with no effect on p16 expression.
